# Supplementary material for: Association between oral microbiome and seven types of cancers in East Asian population: a two-sample Mendelian randomization analysis
Source: Front Mol Biosci. 2023 Nov 21;10:1327893. doi: 10.3389/fmolb.2023.1327893 (PMC10702768; doi:10.3389/fmolb.2023.1327893)
Supplement: Supplementary file 1 [file DataSheet1.ZIP › Supplementary Materials/MR plots of saliva/saliva═╝/Breast cancer/pheno.1336_to_breast cancer_leave_one_out.pdf]

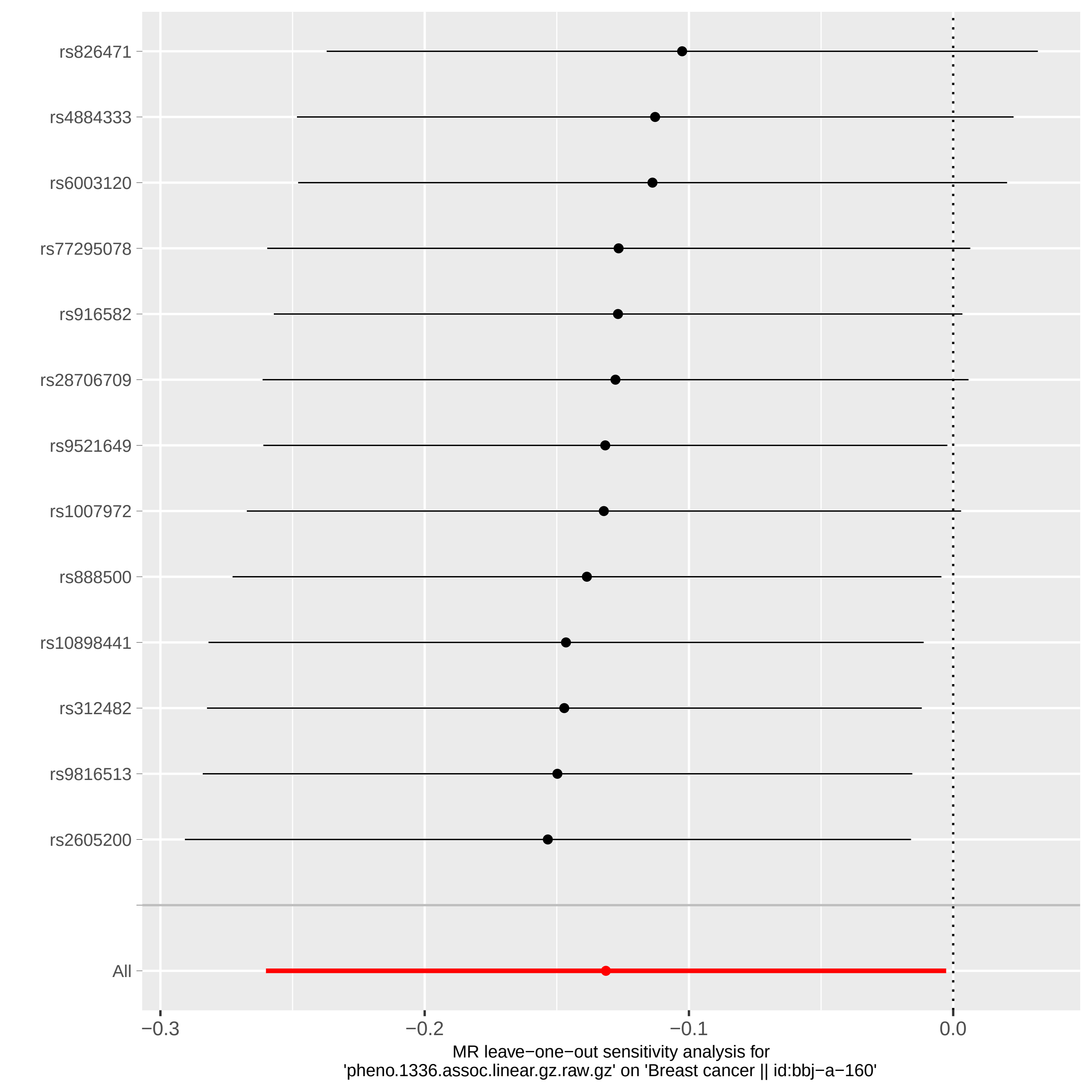

rs826471
rs4884333
rs6003120
rs77295078
rs916582
rs28706709
rs9521649
rs1007972
rs888500
rs10898441
rs312482
rs9816513
rs2605200
All
−0.3
−0.2
−0.1
0.0
MR leave−one−out sensitivity analysis for
'pheno.1336.assoc.linear.gz.raw.gz' on 'Breast cancer || id:bbj−a−160'
